# Soil health improvements under cover crops are associated with enhanced soil content of cytokinins

**DOI:** 10.1111/plb.13743

**Published:** 2024-12-06

**Authors:** I. Perera, A. Kisiala, K. A. Thompson, R. J. N. Emery

**Affiliations:** ^1^ Department of Environmental and Life Sciences Trent University Peterborough Ontario Canada; ^2^ Department of Biology Trent University Peterborough Ontario Canada; ^3^ Trent School of Environment Trent University Peterborough Ontario Canada

**Keywords:** Cereal rye, cover crops, cytokinins, soil health indicators, soil microbial communities, tillage radish

## Abstract

- Cytokinins (CKs) are phytohormones produced by plants and other soil life. including bacteria, fungi, insects, and earthworms. These organisms can release CKs to the soil, which may have positive implications for soil health and plant growth. However, no studies have examined phytohormones as soil health indicators.
- In custom‐designed rhizo‐pots that separated rhizosphere and bulk soils, the cover crops tillage radish and cereal rye were used to manipulate soil health parameters: soil pH, soil organic matter, soil active carbon, soil microbial community diversity, and extracellular enzyme activities involved in C, N and P cycling. Data were compared to impacts of cover crops on CKs that were purified from the complex soil and measured with HPLC‐HRMS/MS.
- From soil we detected free base‐CKs (*trans*‐zeatin (tZ), isopentenyladenine (iP)), riboside‐CKs (RB‐CKs), *cis*‐zeatin riboside (cZR), isopentenyladenosine (iPR) and four methylthiolated CKs: 2‐methylthio‐zeatin (2MeSZ), 2‐methylthio‐zeatin ribosides (2MeSZR), 2‐methylthio‐isopentenyladenine (2MeSiP), and 2‐methylthio‐isopentenyladenine riboside (2MeSiPR). These CK levels were significantly enhanced in cover cropped soil compared to uncultivated soil, and reflect a positive relationship between soil CK profiles and other soil health parameters – notably, between total CK and active C levels and soil microbial community diversity.
- This is the first detailed soil CK analysis and assessment of its potential use as a novel, reliable, short‐term soil health parameter. The increased CK concentrations in cover cropped soils likely reflects the activity levels of soil life (plants, microbes, animals) and provides a rationale to use CKs as tools to evaluate soil health as influenced by agricultural management strategies.

## Graphical abstract

Total soil CK levels correlate with soil health parameters (AC, SMC diversity), indicating CKs may represent a novel indicator of short‐term changes in soil health.

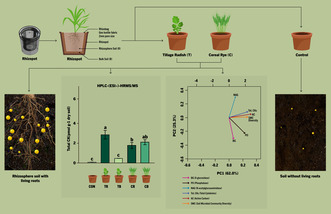

## INTRODUCTION

Soil is a natural, non‐renewable, dynamic ecosystem. It is composed of inorganic matter (rock and mineral particles), organic matter (e.g., plant and animal residues, byproducts of microbial decomposition), living organisms, water, and gases (Pulleman *et al*. 2012; Dazzi & Lo Papa 2022). Soils are considered the largest reservoir of biodiversity on Earth, acting as a substrate that supports plant growth and provides habitat for a wide range of soil‐dwelling organisms, such as microbes, nematodes, earthworms, and insects (Yang *et al*. 2018; reviewed in Doran & Zeiss 2000). Soil‐dwelling microorganisms, collectively described as soil microbial communities (SMC), play an essential role in nutrient cycling in the ecosystem via production of enzymes involved in decomposition of soil organic matter (SOM), nutrient mineralization, N fixation, N‐transformation reactions, etc. (Tregubova *et al*. 2021; Grzadziel *et al*. 2018; Stockdale & Watson 2009; reviewed in Doran & Zeiss 2000).

Soil is a principal feature in most terrestrial ecosystems, including agricultural fields (reviewed in Schoonover & Crim 2015; Stockdale & Watson 2009; reviewed in Doran & Zeiss 2000) and, therefore, maintaining good soil health is crucial for the well‐being of plants and animals, the long‐term sustainability of agricultural production systems, and a healthy environment (Grzadziel *et al*. 2018). Soil health can be defined as the continued capacity of the soil to function as a living ecosystem while maintaining environmental quality and the health of plants, animals, and humans (Doran & Parkin 1994; Doran & Zeiss 2000; Lehman *et al*. 2017; Bonfante *et al*. 2020). Soil health is assessed using a combined set of measurable physiochemical and biological attributes, known as soil health parameters (Prabha et al., 2020). Examples of common soil parameters used in assessing soil health include soil aggregate stability, bulk density, and water‐holding capacity, as well as chemical parameters, including soil pH, soil fertility, SOM, soil organic carbon (SOC) and biological indicators, such as active carbon (AC), abundance and diversity of SMC, respiration, or assays of microbial extracellular enzyme activity (EEA) (Lehman *et al*. 2017; Bonfante *et al*. 2020).

Soil degradation and declines in soil health due to intensive agriculture are major problems that farmers face worldwide (Bedolla‐Rivera *et al*. 2023). One of the recommended management strategies to improve soil health is the adoption of cover crops in cash‐cropping systems (Cover Crops Canada, 2018). Cover crops are living ground covers integrated into crop rotations between main crops or in fallow periods. If not winter‐killed or used for grazing or feed, cover crops are often terminated using manual or using chemical methods before the next main crop phase (Hartwig & Ammon 2002; Fageria *et al*. 2005; Justes 2017). Cover crop species provide a variety of ecosystem services, including protection from soil erosion, reduction of nutrient leaching, suppression of weeds and soil‐borne pathogens, biomass production contributing to residue returns, and if legumes are included, increased atmospheric N_2_ fixation (Belfry *et al*. 2017). Integration of cover crops in cash crop rotations results in enhanced soil health, as illustrated by increases in SOM, SOC, total N content, AC, hydraulic conductivity, infiltration rates, and soil aggregation (Ghimire *et al*. 2019; Nouri *et al*. 2019; Zeynep *et al*. 2019; Chahal & Van Eerd 2020). Soil health improvement by cover crops is of critical significance for plant growth promotion and increased cash crop yields (Congreves *et al*. 2015; Belfry *et al*. 2017).

Phytohormones, are small signalling molecules which are crucial factors involved in plant growth promotion, biomass formation, and other physiological processes in plants throughout their life cycle (Jameson & Song 2016; Smith *et al*. 2017). Phytohormones are produced by living plants but also by soil inhabitants, including bacteria, fungi, insects, nematodes, and earthworms, among others (Palberg *et al*. 2022; Andreas *et al*. 2020; Aoki *et al*. 2019; reviewed in Wong *et al*. 2015; reviewed in Stirk & van Staden 2010). Plants and microorganisms can release endogenously produced phytohormones into their immediate environment, such as soil (Bean *et al*. 2021; Palberg *et al*. 2022; Jorge *et al*. 2019; reviewed in Wong *et al*. 2015; Kisiala *et al*. 2013; reviewed in Stirk & van Staden 2010; Timmusk *et al*. 1999; Phillips & Torrey 1972), which can have positive implications for soil health and plant growth. However, no studies have yet examined phytohormones as potentially important soil health indicators.

Cytokinins (CKs) are a group of phytohormones responsible for plant growth promotion and improvement of crop yields (Stirk & van Staden 2010; Jameson & Song 2016; Goh *et al*. 2019; High *et al*. 2019; Jorge *et al*. 2019). They are evolutionarily conserved among distinct groups of organisms, and their presence has been detected in representatives of all kingdoms of life (Spíchal 2012; Aoki *et al*. 2019). Two major CK biosynthetic pathways are known: the tRNA degradation pathway, which is present in all organisms, and the de novo pathway which is best known in gymnosperms and angiosperms as well as some microorganisms (Spíchal 2012, Hluska et al 2021). These two pathways lead to characteristic forms of CKs with tRNA degradation notably producing *cis*‐isomers and the de novo pathway leading to trans‐isomers of CK.

The chemical structure of CKs contains an adenine derivative with an isoprenoid or aromatic side chain at N^6^ position. Based on configuration of the molecule, CKs can be either active or act as inactive precursors or conjugates. Free base CKs, including isopentenyladenine (iP), *trans*‐zeatin (tZ), *cis*‐zeatin (cZ), and dihydrozeatin (DHZ), are considered the most biologically active CKs (Kisiala *et al*. 2019; Kieber & Schaller 2018; Jameson & Song 2016; Spíchal 2012; Romanov & Schmülling 2021). The most common CK conjugates are glucoside‐CKs, riboside‐CKs, and nucleotide‐CKs. Another, less well known CK group, the methylthiolated CKs (2MeS‐CKs), are derivatives of Z and iP with a thiol group (–SH) at position 2 of the adenine ring, and are derived through the tRNA degradation pathway (reviewed in Gibb *et al*. 2020).

In plants, CKs are involved in the regulation of a wide spectrum of development processes, including shoot and root growth, control of shoot apical dominance and branching, leaf expansion, development of chloroplasts and chlorophyll production, delaying leaf senescence, and reproductive development (Bartrina *et al*. 2017; Cortleven *et al*. 2019; Kieber & Schaller 2014; reviewed in Stirk & van Staden 2010). Furthermore, CKs mediate plant responses to abiotic environmental factors, such as nutrient availability, osmotic potential, heat, salinity, and drought (reviewed by High *et al*. 2019; Jorge *et al*. 2019; Pavlů *et al*. 2018), in addition to improving plant immunity against pathogen infections (Gupta *et al*. 2020). Plant roots are potentially extrusive sites of CK biosynthesis, given their high surface area and ability of CK to pass through membranes, which facilitates exchange between plants and soil. Hence, plant roots can release significant levels of CKs into their environment (Stirk & van Staden 2010).

Furthermore, the presence of active CKs has been reported in *in vitro* cultures of diverse microorganisms. Kisiala *et al*. (2013) identified the ability of N_2_‐fixing, symbiotic rhizobia strains to produce 25 forms of CK, including bioactive forms. Studies have also reported the ability of *Methylobacterium* spp. to produce different CK forms *in vitro* (Palberg *et al*. 2022), and when inoculated in lentil (Jorge *et al*. 2019). Bean *et al*. (2021) identified symbiotic fungi, *Trichoderma* spp., able to produce CKs; while Morrison *et al*. (2015) reported the prevalence of CK production derived from the tRNA degradation pathway in 20 temperate forest fungi of differing nutritional modes. Other potential sources of CKs in soil are decomposing plant matter and other CK‐producing soil‐dwelling organisms, such as earthworms, nematodes, and insects (Stirk & van Staden 2010; Wong *et al*. 2015; Andreas *et al*. 2020).

Despite the extensive knowledge of CK production by non‐plant organisms, their biological roles and potential benefits are largely undefined. However, CKs have ample opportunity to feedback and impact plant and non‐plant organisms alike once they are outside of living organisms and in the soil matrix. Studies on soil CK profiles are limited, resulting in poor understanding of the potential role of CK functioning in the soil ecosystem. To date, only a small number of studies have reported the occurrence of CKs in soil (Van Staden *et al*. 1976; Nieto & Frankenberger 1989; High *et al*. 2019). High *et al*. (2019) identified iP and tZ‐type CKs in soils and described the positive impact of earthworms on soil CK content. Furthermore, in an early study, Van Staden *et al*. (1976) showed the combined effects of living roots and rhizosphere microorganisms, including *Rhizobium* sp. and mycorrhizal fungi (*Lycoperdon* sp. and *Scleroderma* sp.), to enhance levels of zeatin (Z) and zeatin riboside (ZR)‐like activity in soil using an indirect bioassay with soybean callus.

We carried out a greenhouse experiment with two non‐leguminous cover crop species—cereal rye (*Secale cereale* L.) and tillage radish (*Raphanus sativus* L. var. *longipinnatus*)—to identify the impact of cover crops on soil CK profiles and to study relationships between traditional soil health parameters (e.g., pH, SOM, AC, microbial EEAs, SMC diversity, microbial C use patterns) and soil CK levels. For both cover crop species, the soil health parameters and soil CK profiles were assessed in two soil zones—rhizosphere and bulk soil. We purified soil CKs using solid phase extraction and identified and quantified picomolar levels of free base CKs (iP, tZ), RB‐CKs (cZR, iPR), and 2MeS‐CKs (2MeSZ, 2MeSiP, 2MeSZR, 2MeSiPR) present in the soil matrix using High‐Resolution Accurate‐Mass QExactive Orbitrap tandem mass spectrometry (HPLC‐HRMS/MS). As such, this study presents the most comprehensive analysis of CK profiles in soil to date. Our study revealed that cover crops improve soil health parameters, such as SOM, AC, SMC diversity, and EEAs, while at the same time, they strongly enhance soil CK profiles. Importantly, soil CK levels were positively correlated with selected soil health parameters, such as AC, SMC diversity, and EEAs. This demonstrates that characterization of soil CK profiles can be a useful new soil health indicator that can be used to monitor short‐term impacts of agricultural management practices.

## MATERIAL AND METHODS

### Soil and plant material

Soils were collected for the greenhouse pot experiment from two different locations at the Trent Experimental Farm (TEF) at Trent University in Peterborough, Ontario, Canada (44°21′45.9″N, 78°16′55.1″W) in 2019. The particle size distribution of clay, silt, and sand, measured using a standard buoyancy hydrometer (Fisher Scientific), indicated that the two soils had different textures, namely loam (L) (12% clay, 48% silt, 40% sand), and sandy loam (SL) (12% clay, 24% silt, 64% sand). Soils were air‐dried and passed through a 2‐mm sieve to remove stones and plant debris before seed potting.

Seed of two cover crop species (obtained from Sunderland Co‐op, Sunderland, Canada) were used in the experiment: tillage radish (*Raphanus sativus* L. var. *longipinnatus*) and cereal rye (*Secale cereale* L.). These are non‐legumine cover crops that fall into the categories of *Brassica* spp. (tillage radish) and Poaceae spp. (cereal rye). These two cover crop types have different root structures, where tillage radish has a tuber with a long taproot, and cereal rye has a shallow, fibrous root system.

### Greenhouse pot experiment

**Fig. 1:**
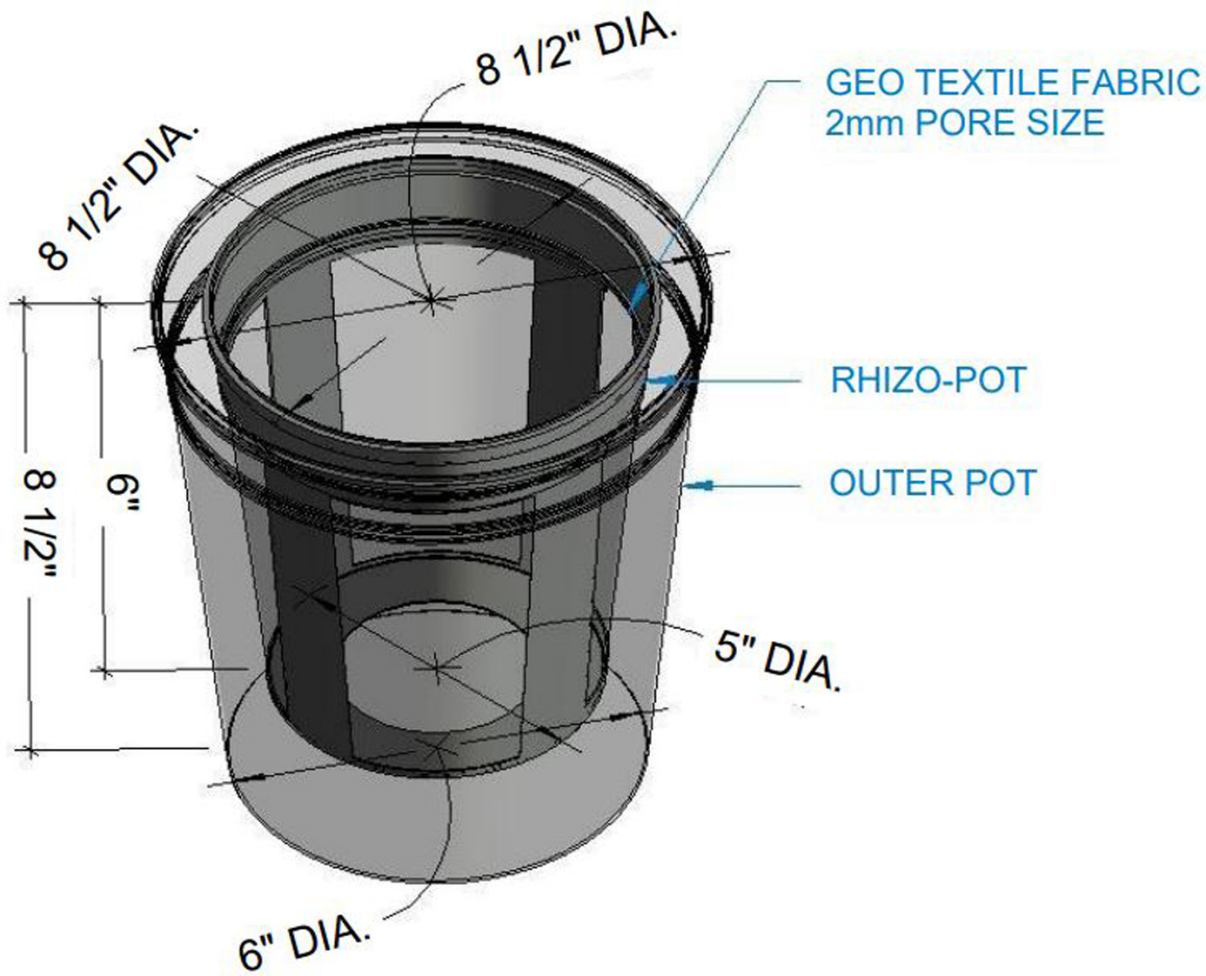
Schematic diagram of pots used in the greenhouse experiment. A single pot was fitted with a small pot inside and called a ‘rhizo‐pot’. The rhizo‐pots were custom‐designed using geotextile fabric with a 2‐μm pore size to allow water, nutrients, and microbes to pass between the rhizosphere and bulk soil zones while restricting root growth from the rhizosphere to bulk zone.

The greenhouse trial was established to separately analyse the impacts of the two cover crops on soil health parameters and soil CK levels in the rhizosphere and bulk (external to the rhizosphere) soils. Customized pots were designed to help separate rhizosphere soil and bulk soil at the end of experiment. A single pot was comprised of a small pot, called the ‘rhizo‐pot’, that was built using geotextile fabric to allow movement of water, nutrients, and microbes between soil zones while restricting root growth; these pots were placed into the larger, ‘outer pots’ (Fig. 1). Combined pots were filled with soil and placed in the greenhouse in a randomized block design. Both soil types; Loam (L) and Sandy loam (SL) were used in this trial to study the impact of soil texture on CK levels and soil health parameters. Baseline soil, used as control in this study, refers to the soil before any potting.

Eight pots were filled with ca. 1 L of the SL soil and used to plant tillage radish (4 pots) and cereal rye (4 pots). Due to limitations in the amount of SL soil collected during the previous fall season, only three pots of tillage radish were grown in the SL soil. After emergence, tillage radish seedlings were thinned to four plants per pot, and cereal rye seedlings were thinned to six plants per pot. Plants were watered daily with tapwater and harvested after 3 months. Following harvest, soil from the two compartments (‘rhizosphere’ and ‘bulk’) were collected separately and stored at −20°C until analysis.

### Soil pH and soil C analysis

For soil pH, soil solutions were prepared by mixing soil and distilled water in a 1:2 ratio, and measurements taken with a pre‐calibrated pH meter (Mettler‐Toledo) (Hendershot et al. 2008). Five technical replicates were completed for compartment × cover crop. The compartment refers to the Bulk and Rhizosphere soils. The SOM content was measured in five technical replicates of soils (5 g) from each soil compartment × cover crop sample as loss on ignition (LOI) at 5550°C for 4 h in a muffle furnace. Mass of the soil before and after ignition was recorded, and percentage difference in weight was taken as the SOM content [%]. AC, the labile fraction of SOC which is readily used by SMCs, was assessed using the KMnO_4_ method (Weil *et al*. 2003). KMnO_4_ oxidizes simple carbohydrates, amino acids, amines and amides, and C compounds that have a hydroxyl group (C‐OH), ketone (R2C=O), carboxyl (−COOH), and aliphatic compounds, which are preferred substrates of the heterotrophic SMC. The absorbance of C oxidized by KMnO_4_ was obtained at 550 nm with an Epoch™ microplate spectrophotometer (BioTek Instruments, Germany). A triplicate standard curve of KMnO_4_ with a dilution series of known concentrations (0.05 M, 0.01 M, 0.015 M. and 0.02 M) was prepared and plated on the same microplate to determine AC concentrations in the soil samples.

### Community‐level physiological profiling (CLPP) and SMC diversity

To assess the patterns of C source utilization by SMCs in rhizosphere and bulk soils under each of the cover crops/soil types, CLPP was carried out (Weber & Legge 2010). 96‐well plates (Biolog EcoPlates™) consisting of 31 different C sources in triplicate with a blank (water) were used for the assay. The wells in the BIOLOG plates contain a C source and tetrazolium violet, which is a redox dye indicator. When the inoculated microbial communities utilize these C sources and respire, the tetrazolium dye is reduced to formazan, resulting in a colour change (colourless to pink/violet). The ability of the soil microbial communities to utilize the different C sources was measured based on the colour development. Prior to the assay, soils were pre‐incubated in Petri dishes covered with aluminium foil in the dark at room temperature (25°C) for 7 days. The supernatant of the soil solution (10 g pre‐incubated soil in 90 mL 0.85% NaCl solution) was diluted to 10^−3^ before inoculation into the wells of the BIOLOG plates (150 μL aliquot in each well). The BIOLOG plates were incubated in the dark at room temperature (20°C) for 7 days.

Absorbance readings were obtained with an Epoch™ microplate spectrophotometer (BioTek Instruments, Germany) at 590 nm every 24 h for 168 h over the 7‐day incubation period. Since maximum absorbance readings (OD_590_) were obtained at 168 h, a single time point absorbance at 168 h was used in the data analysis. In addition, the absorbance readings obtained by CLPP (at 168 h) were used to calculate SMC diversity using the Shannon diversity index (Weber & Legge 2010; D'Acunto *et al*. 2018).

### Microbial extracellular enzyme activity (EEA)

Microbial EEAs, including ꞵ‐glucosidase (BG), phosphatase (PO), and N‐acetylglucosaminidase (NAG), were determined in rhizosphere and bulk soils under cover crops (Jackson et al., 2013). The assays were conducted by incubating 150 μL soil solution (5 g of soil in 5 mL 50 mM acetate buffer) at room temperature (20°C) with 150 μL *p*‐nitrophenyl (*p*NP) linked substrates: *p*NP‐ꞵ‐D‐glucopyranoside (5 mM, 1 h), *p*NP‐ꞵ‐N‐acetylglucosaminide (2 mM, 2 h), or *p*NP‐phosphate (5 mM, 1 h), respectively, for the enzymes: ꞵ‐glucosidase (BG), N‐acetylglucosaminidase (NAG), and phosphatase (PO), followed by absorbance readings using an Epoch™ microplate spectrophotometer (BioTek Instruments) at 410 nm. Soil EEAs (μM h g^−1^ dry soil) were calculated using standard curves prepared with serial dilution of known standards containing 0.025–1 mM *p*NP.

### Analysis of soil CK profiles using HPLC‐HRMS/MS

The CK analyses were conducted to determine the diversity and abundance of soil CK profiles in rhizosphere and bulk soils under each cover crop/soil type. Thirty‐two CK forms were investigated, and any individual CKs detected were assessed separately and as part of their functional group category (free base CKs, riboside CKs, and methylthiolated CKs) and the functional categories grouped together as soil total CKs (i.e., all active CKs with inactive conjugates). Prior to analysis, dry soil samples (1.5 g) were suspended in ice‐cold 50% acetonitrile (ACN) solution, spiked with isotopically labelled internal CK standards (10 ng): [^2^H_5_]ZR, [^2^H_3_]DZR, [^2^H_6_] iPR, [^2^H_3_]DZ, [^2^H_6_]iP, [^2^H_6_]2MeSZ, [^2^H_6_]2MeSZR, [^2^H_6_]2MeSiP, [^2^H_6_]2MeSiPR, [^2^H_5_]ZOG, [^2^H_7_]DHZOG, [^2^H_5_]ZROG, [^2^H_7_]DHZROG, [^2^H_5_]Z9G, [^2^H_5_]DHZ9G, and standards (50 ng): gibberellin (GA_1_), jasmonic acid, salicylic acid and 160 ng indole‐3‐acetic acid (OlChemIm, Olomouc, Czech Republic), and ground using a Retsch MM400 ball mill (Retsch, Haan, Germany) with two zirconium oxide beads (Comeau Technique, Vaudreuil‐Dorion, Canada). The homogenized soil samples were purified using HLB cartridges (Canadian Life Sciences, Peterborough, Canada) according to a modified protocol (Šimura *et al*. 2018). Quantification of CKs was carried out in Parallel Reaction Monitoring (PRM) mode using Orbitrap QExactive ‐ HRMS) (Thermo Scientific, San Jose, USA) coupled with a Dionex Ultimate3000 HPLC system (Kisiala *et al*. 2019).

Chromatographic separation of CKs was achieved on a Kinetex C18 column (2.1 i.d. ×50 mm, 2.6 μm particle size; Phenomenex, Torrance, USA) with a SecurityGuard C18 guard cartridge (Phenomenex.), using an Ultimate 3000 UHPLC (Thermo Scientific) equipped with an HPG‐3400RS dual pump and a WPS‐3000 auto sampler. The UHPLC column was operated at room temperature (∼22°C). A flow rate of 0.5 mL min^−1^ was applied with a binary gradient of water (A) and acetonitrile (B), both with 0.08% glacial acetic acid. The initial gradient (5% B), was held for 0.5 min, increased linearly to 45% B over 4.5 min, increased again to 95% B over 0.1 min, and held at 95% B for 1 min, before returning to initial conditions for 2 min of column re‐equilibration. For the heated electrospray ionization source, temperatures of the auxiliary gas heater and capillary were 450°C and 300°C, respectively, and the spray voltage was 3.9 kV. Sheath, auxiliary, and sweep gases were operated at 30, 8, and 0 (arbitrary units), respectively. The S‐lens RF level was 60. The applied HESI‐II conditions yielded <5% in‐source fragmentation of the CKs. Acquisition was performed in positive ion mode, and data were acquired in time‐scheduled PRM events. Targeted scan windows were used to enhance the ion signal intensity for each CK analyte. To determine compound scanning start and end times, CK metabolites were first detected using FS mode with a mass range of m/z 150 to 550. All FS and PRM data were acquired at resolution of 35 000 fwhm (full width at half‐maximum) at m/z 200. Further PRM parameters included an automatic gain control (AGC) of 3 × 106 and a maximum injection time (IT) of 128 ms. The precursor isolation window width was m/z 1.2. The normalized collision energy (NCE) was individually optimized for each compound in stepwise increments, where at least 10% of the unfragmented precursor ion was retained. Limits of detection are given in Kisiala *et al*. (2019) and ranged from 0.1 to 8.0 fmol, depending on the compound. Obtained phytohormone data were quantified using Xcalibur 3.0.63 software (Thermo Scientific) using the isotope dilution method based on the recovery of endogenous compounds and recovery of internal standards. CK standards were *trans*‐isomers which were used to quantify both endogenous *cis‐* and *trans*‐CKs.

### Statistical analysis

All statistical analyses were conducted using the R Studio software (2021 R Studio: Integrated Development Environment for R. R Studio, PBC, Boston, MA, USA). Soil health parameters and CK concentration were compared between the two non‐plant control soil types (L and SL soils) collected from Trent Experimental Farm, using Student *t*‐tests. One‐way ANOVA was conducted to test for differences in soil CK levels, soil health parameters (pH, SOM, AC, EEA), and SMC diversity between control soil and cover‐cropped soils of both types (L soils—TR, TB, CR, CB; SL soils—TR and TB) (Table 1) with a generalized linear model. Mean separation between control soil and cover‐cropped soils was determined using Fisher's Least Significant Difference test (LSD), at significance of *P* ≤ 0.05. Data normality was checked using a Shapiro‐Wilks test, and homoscedastic assumptions were checked using Bartlett's test. Significant differences among and between least‐square means and two‐sample *t*‐tests were determined by *p*‐values, with *P* < 0.05 unless otherwise stated.

**Table 1:** Abbreviations used to denote different treatments and soil types.

abbreviations	description
L	Loam soil
SL	Sandy loam soil
TR	Tillage radish‐ Rhizosphere
TB	Tillage radish‐ Bulk
CR	Cereal rye‐ Rhizosphere
CB	Cereal rye‐ Bulk

For CLPP, the data were corrected prior to statistical analysis by subtracting OD values of blanks from the mean OD value of C substrates, and negative values were set to zero (Feigl *et al*., 2017). Next, data were normalized by dividing OD values by average well colour development (AWCD) within a single Ecoplate™. Normalized data at one time‐point (168 h) were used to determine similarities and differences in SMC C‐source utilization patterns and SMC diversity between soil zones and between cover crops and soil types. The wells in the BIOLOG plates contain a C source and tetrazolium violet, which is a redox dye indicator. When the inoculated soil microbial communities utilize these C sources for respiration, the tetrazolium dye reduces to formazan, resulting in a colour change (colourless to pink/violet). The ability of soil microbial communities to utilize the different C sources was measured based on colour development. To visually demonstrate the multiple relationships of soil health parameters and the CK profiles between cover crop type and soil zones in cover‐cropped L soils, principal components analysis (PCA) was conducted. Since the number of variables (*P* = 31 different C sources; Table S1) was greater than the number of samples (*N* = 22 soil samples) (*P* > *N*), we conducted a sparse PCA (sPCA), which reduces the dimensionality of the dataset by adding sparsity to the input variables, to explain the variance among the many variables' relationships and to build a two‐dimensional plot for visual cluster analyses using the “sparse principal component analysis HJ biplot” package in R software. Furthermore, the CLPP data were used to calculate SMC diversity using the Shannon‐Weaver diversity index in R Studio software with the package “vegan.” The matrix used for the sPCA analysis consisted of a total of 16 data points/samples (N), which included cover‐cropped L soil samples (*N* = L soil: TR (4), TB (4), CR (4), CB (4)), and six variables (P) (*P* = AC, SMC diversity, NAG activity, BG activity, PO activity, Total CK). A scree plot was examined for breaks, and PC components with eigen values ≥1 (PC1 and PC2) were retained for the two‐dimensional plot for visual analysis.

## RESULTS

### Analysis of soil health characteristics in control, uncultivated soil

The SL control soil had significantly higher SOM and AC content compared to the L control soil (Table 2). No significant differences were observed in soil pH, SMC diversity or total soil CK levels between the two control soil types (Table 2).

**Table 2:** Comparison of characteristics of loam and sandy loam soils collected from the Trent Experimental Farm, Peterborough, ON.

	loam soil	sandy loam soil
pH	7.67 ± 0.20	7.45 ± 0.11
SOM	5.14 ± 0.05*	5.85 ± 0.04
AC	316.19 ± 32.03*	674.27 ± 2.89
SMC diversity	1.82 ± 0.42	2.15 ± 0.08
Total CK	0.09 ± 0.01	0.16 ± 0.07

Data (mean ± SE) were tested using a two‐sample *t*‐test for pH, soil organic carbon (%) (SOM), active carbon (AC) (ppm), soil microbial community (SMC) diversity (Shannon diversity index), and total CK (pmol g^−1^ dry soil). Mean (*n* = 3) ± SE followed by an asterisk (*) in a row are significantly different between soil types (*P* < 0.05).

### Analysis of soil health characteristics in cover‐cropped soils

#### Soil pH and soil carbon

**Fig. 2:**
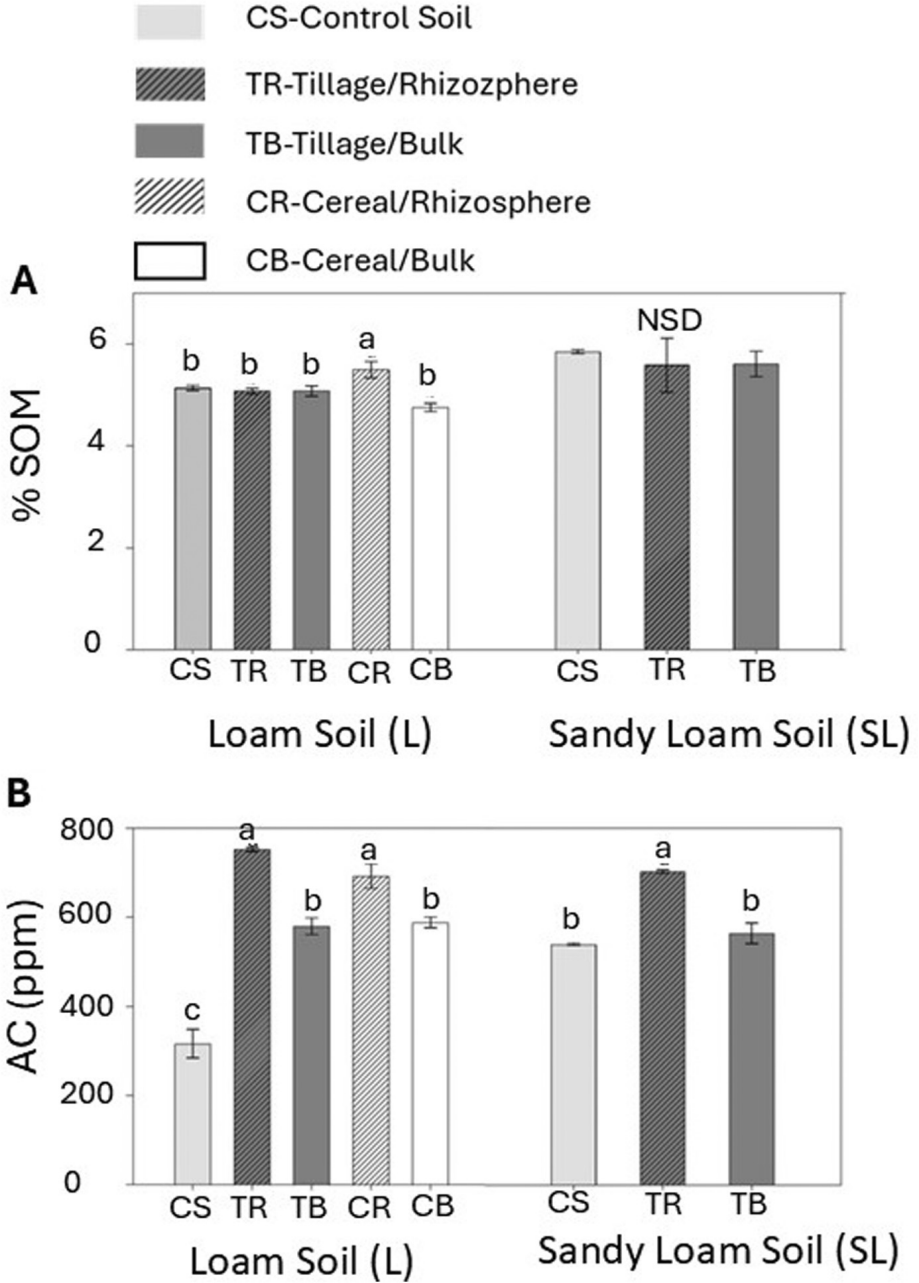
The effect of cover crops on **(A)** soil organic matter (SOM), **(B)** active carbon (AC) levels compared to control, uncultivated soils. ANOVA was used to compare metrics between crops and soil zones within loam soil and sandy loam soils CS (control soil); tillage radish, rhizosphere soil (TR); tillage radish, bulk soil (TB); cereal rye, rhizosphere soil (CR); cereal rye, bulk soil (CB). Values are mean ± SE (*n* = 3), different letters above columns indicate significant differences among treatments (Fisher's least significant difference test, *P* ≤ 0.05).

No significant differences in pH were observed between the cover‐cropped soils and both control soil types (data not shown). The levels of SOM were significantly higher only in L‐CR soils compared to the control L soil. No differences in SOM were found among the cover‐cropped SL soils and the respective control soil (Fig. 2A). All cover‐cropped L soils had higher levels of AC compared to control soils, while cover‐cropped SL soils had higher AC levels than control soils only in the rhizosphere zone (*P <* 0.05) (Fig. 2B). In both L and SL cover‐cropped soils, AC levels were significantly higher in rhizosphere zones compared to their paired respective bulk soils (Fig. 2B).

#### Diversity of soil microbial community (SMC) and community‐level physiological profiling (CLPP)

**Fig. 3:**
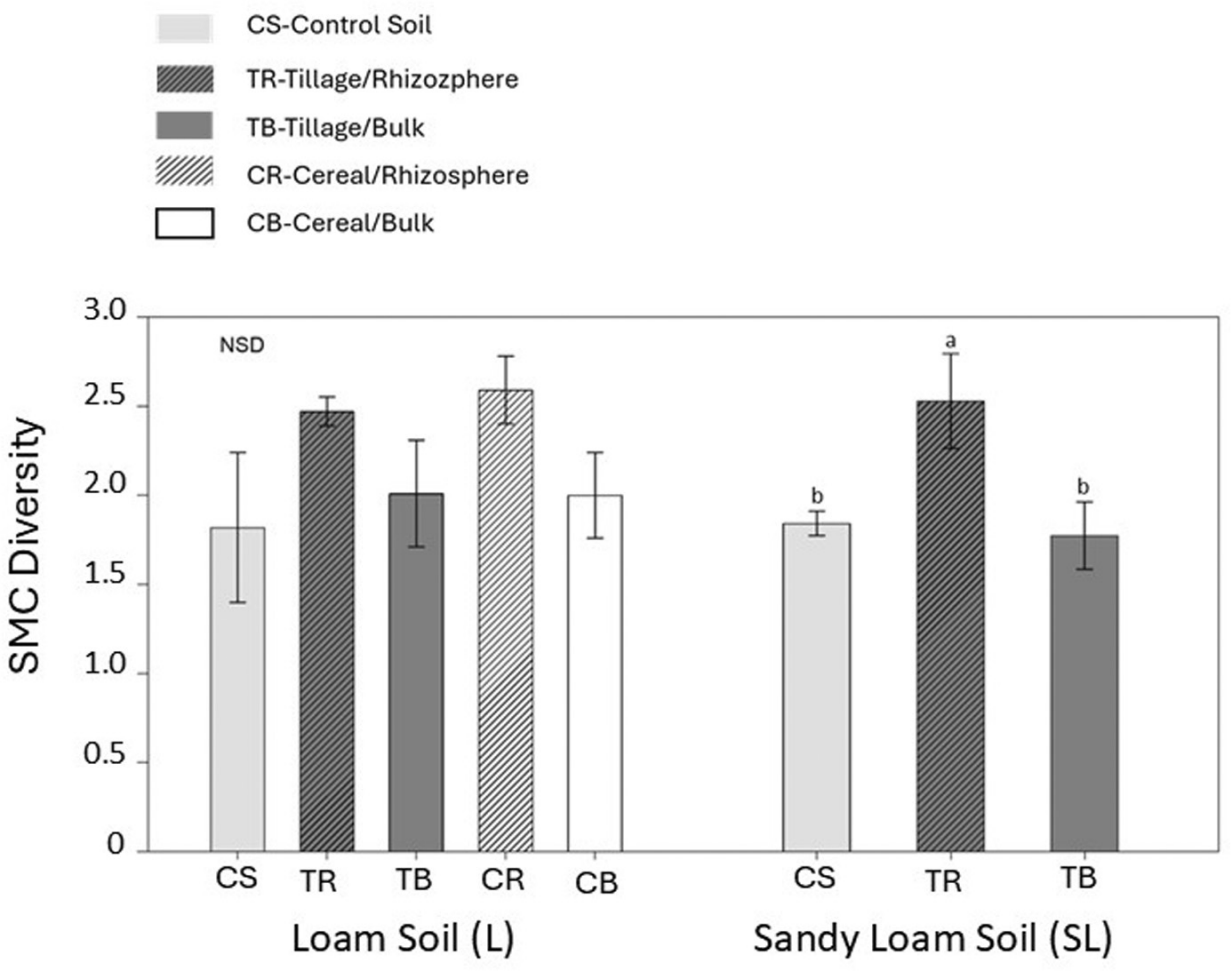
Effects of cover crops on soil microbial community (SMC) diversity, compared to control, uncultivated soils. CS (control soil); tillage radish, rhizosphere soil (TR); tillage radish, bulk soil (TB); cereal rye, rhizosphere soil (CR); cereal rye, bulk soil (CB). The wells in the BIOLOG plates contain a C‐source and tetrazolium violet, a redox dye indicator. When inoculated soil microbial communities utilize these C sources for respiration, the tetrazolium dye reduces to formazan, resulting in a colour change (colourless to pink/violet). The ability of soil microbial communities to utilize different C sources was measured based on colour development. ANOVA was used to compare treatments separately in loam and sandy loam soils. Values are mean ± SE (*n* = 3), different letters above columns indicate significant differences among treatments (Fisher's least significant difference test, *P* ≤ 0.05).

In SL soils, the diversity of the C‐source utilizing SMC was significantly higher in TR compared to the control soil, while there were no significant differences in diversity between any type of cover‐cropped or control L soils (Fig. 3).

**Fig. 4:**
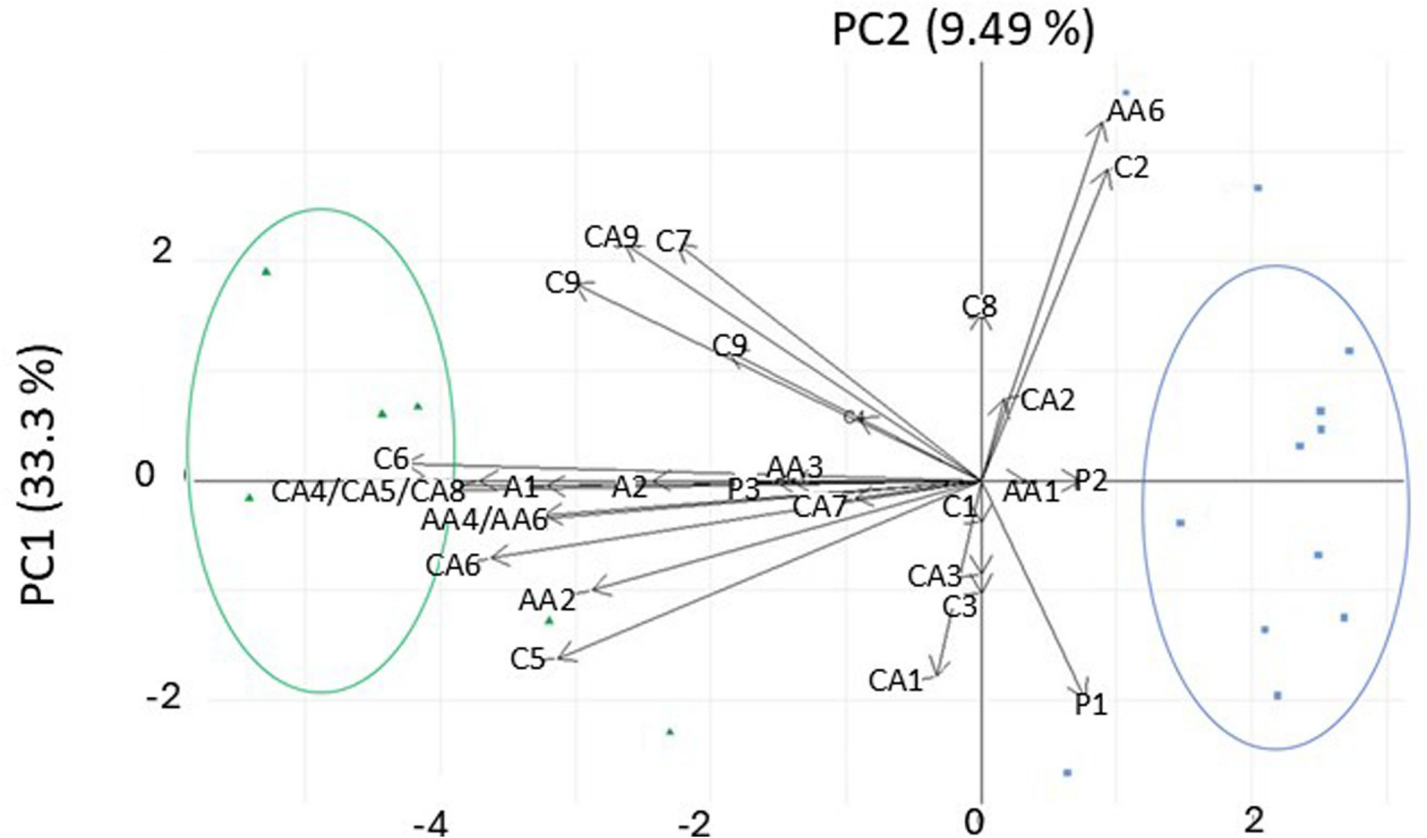
A 2D representation of PC1 and PC2 derived from sparse PCA analysis of normalized absorbance readings (590 nm) of BiologEcoPlates obtained as a result of substrate (30 carbon sources) utilization by soil microbial communities present in sandy loam rhizosphere and bulk soil under tillage radish. Blue squares are scores for rhizosphere soil samples, while green triangles are scores for bulk soil samples. Vector loadings indicate the 31 different carbon sources: carbohydrates (C1‐C10), carboxylic acids (CA1‐CA9), amines and amides (A1 and A2), amino acids (AA1‐AA6), and polymers (P1‐P3). The two ellipses represent the two clusters: green circle illustrates cluster of bulk soil samples, blue circle illustrates cluster of rhizosphere soil samples. A1‐ Phenylethylamine; A2‐ Putrescine; AA1‐ L‐Arginine; AA2‐ L‐Asparagine; AA3‐L‐Phenylalanine; AA4‐ L‐Serine; AA5‐ L‐Threonine; AA6‐ Glycyl‐L‐glutamic acid; C1‐ Pyruvic acid methy ester; C2‐ D‐Cellobiose; C3‐ Alpha‐D‐lactose; C4‐ Beta‐methyl‐D‐glucoside; C5‐ D‐Xylose; C6‐ i‐Erythritol; C7‐ D‐Mannitol; C8‐ N‐Acetyl‐D‐glucosamine; C9‐ Glusose‐1‐phosphate; C10‐ D,L‐a‐Glycerol phosphate; CA1‐ D‐Glucosaminic acid; CA2‐ D‐Galactonic acid y‐ Lactone; CA3‐ D‐Galacturonic acid; CA4–2‐Hydroxy benzoic acid; CA5–4‐Hydroxy benzoic acid; CA6‐ Gamma‐amino butyric acid; CA7‐ Itaconic acid; CA8‐ alpha‐keto‐butyric acid; CA9‐ alpha‐keto‐butyric acid; P1‐ Tween 40; P2‐ Tween 80; P3‐ Alpha‐cyclodextrin.

For the sPCA, which was carried out to visualize C‐source utilization ability of the SMC, CLPP data points of both L soil zones under tillage radish (*n* = 4 (TR) + 4 (TB)) and cereal rye (*n* = 4 (CR) + 4 (CB)), both SL soil zones under tillage radish (*n* = 3 (TR) + 3 (TB)), and all soil samples (L, *n* = 16 + SL, *n* = 6) were taken to create three matrices (*P* × *n*) separately, that represent utilization of 31 C sources in cover‐cropped L soil (31 × 16), cover‐cropped SL soil (31 × 6), and in total soil samples (31 × 22), and generated three sPCA biplots (not shown). After visualization of the sPCA biplots, SL cover‐cropped soils separated out on the biplot (Fig. 4). PC1 accounted for 33.3% of the dataset variance, mainly as SMC utilization of polymers (P2 and P3), carboxylic acids (CA4, CA5, CA6, CA7 and CA8), amino acids (AA1, AA2, AA3, AA4, AA5), and all amines and amides (A1 and A2) were significant loading factors on PC1, while PC2 axis accounted for 9.49% of the dataset variance, and SMC utilization of different carbohydrate types (C1, C2, C3, C4, C6, C7, C9, C10; Table S1, Fig. 4) were significant loading factors. The C source utilization patterns of SMC in rhizosphere and bulk soils showed remarkable separation in the sPCA biplot (Fig. 4).

### Assays of microbial extracellular enzyme activity (EEA)

In L soils, NAG activity was significantly different between the two cover crops. The highest NAG activity was in bulk soil under cereal rye (Table 3). No significant differences were observed in BG and PO activity among any type of cover‐cropped L soils, and there were no significant differences in any EEAs among cover‐cropped SL soils (Table 3).

**Table 3:** Extracellular enzyme activities (μM h g^−1^ dry soil) of ꞵ‐glucosidase (BG), N‐acetylglucosaminidase (NAG), phosphatase (PO) in rhizosphere (TR) and bulk (TB) soil under tillage radish (loam and sandy loam), and rhizosphere (CR) and bulk (CB) soils under cereal rye (loam).

	loam Soil				sandy loam soil	
	TR	TB	CR	CB	TR	TB
BG	0.28 ± 0.04	0.28 ± 0.02	0.25 ± 0.04	0.18 ± 0.01	0.18 ± 0.03	0.23 ± 0.02
NAG	0.14^b^ ± 0.01	0.11^b^ ± 0.01	0.14^ab^ ± 0.01	0.19^a^ ± 0.01	0.10 ± 0.01	0.11 ± 0.01
PO	0.74 ± 0.07	0.60 ± 0.08	0.60 ± 0.02	0.47 ± 0.02	0.43 ± 0.01	0.60 ± 0.12

Comparisons between treatments within loam soil and sandy loam soils were done separately using one‐way ANOVA and a two‐sample *t*‐test, respectively. Means (*n* = 3) ± SE followed by different letters in a row are significantly different (LSD test, *P* < 0.05).

#### Soil cytokinin (CK) profiles

**Fig. 5:**
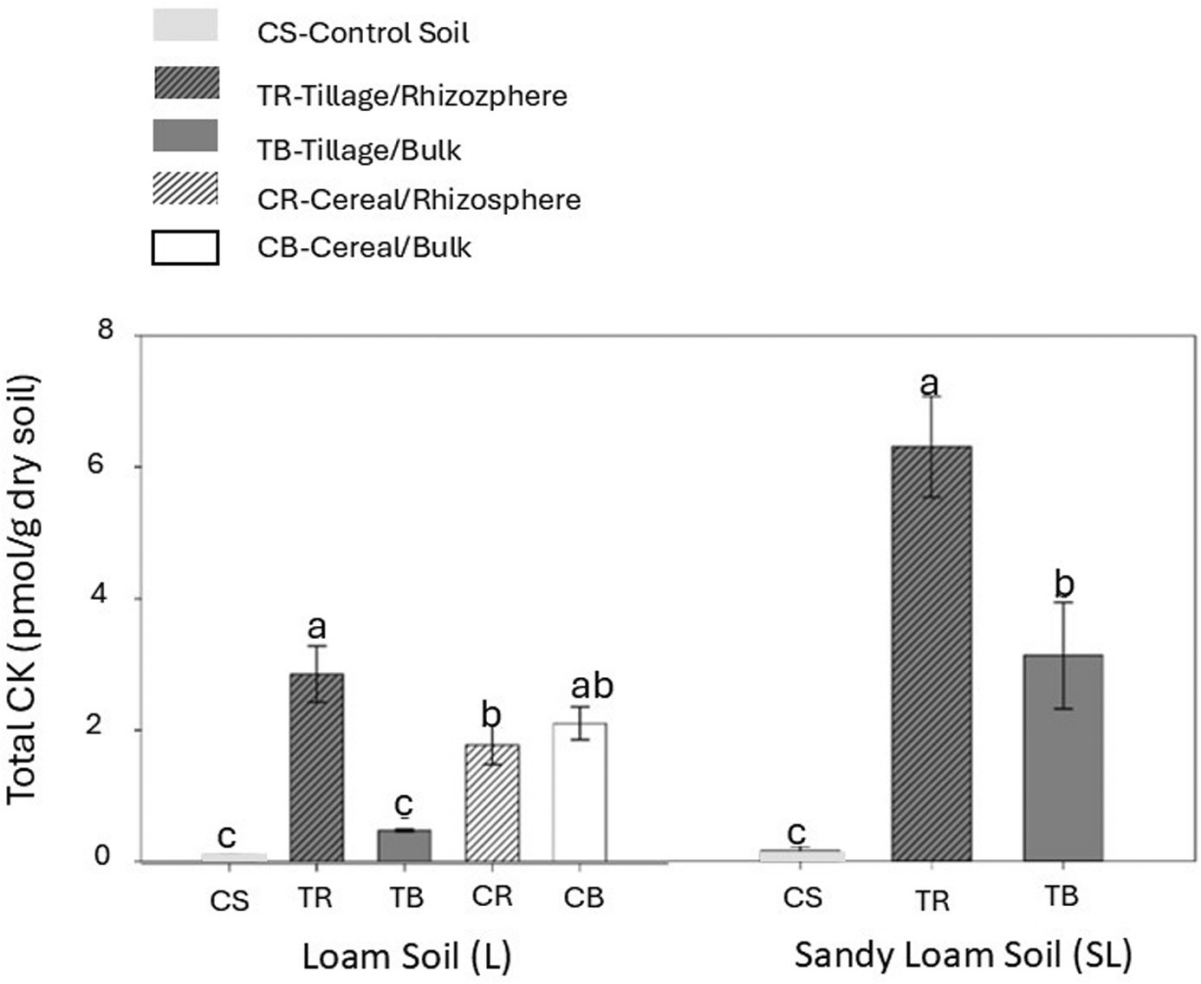
Effect of cover crops on total CK concentrations (pmol g^−1^ dry weight) compared to control, uncultivated soil (CS). Values are mean ± SE (*n* = 3), different letters above columns indicate significant difference among treatments within one soil type (Fisher's least significant difference test, *P* ≤ 0.05). control soil (CS); tillage radish, rhizosphere soil (TR); tillage radish, bulk soil (TB); cereal rye, rhizosphere soil (CR); cereal rye, bulk soil (CB).

The presence of both cover crops significantly increased total CK levels compared to control soils in both rhizosphere and bulk zones of L and SL soils (Fig. 5). Rhizosphere soils under tillage radish had significantly higher levels of total CKs compared to their bulk soil counterparts in both soil types (Fig. 5).

**Fig. 6:**
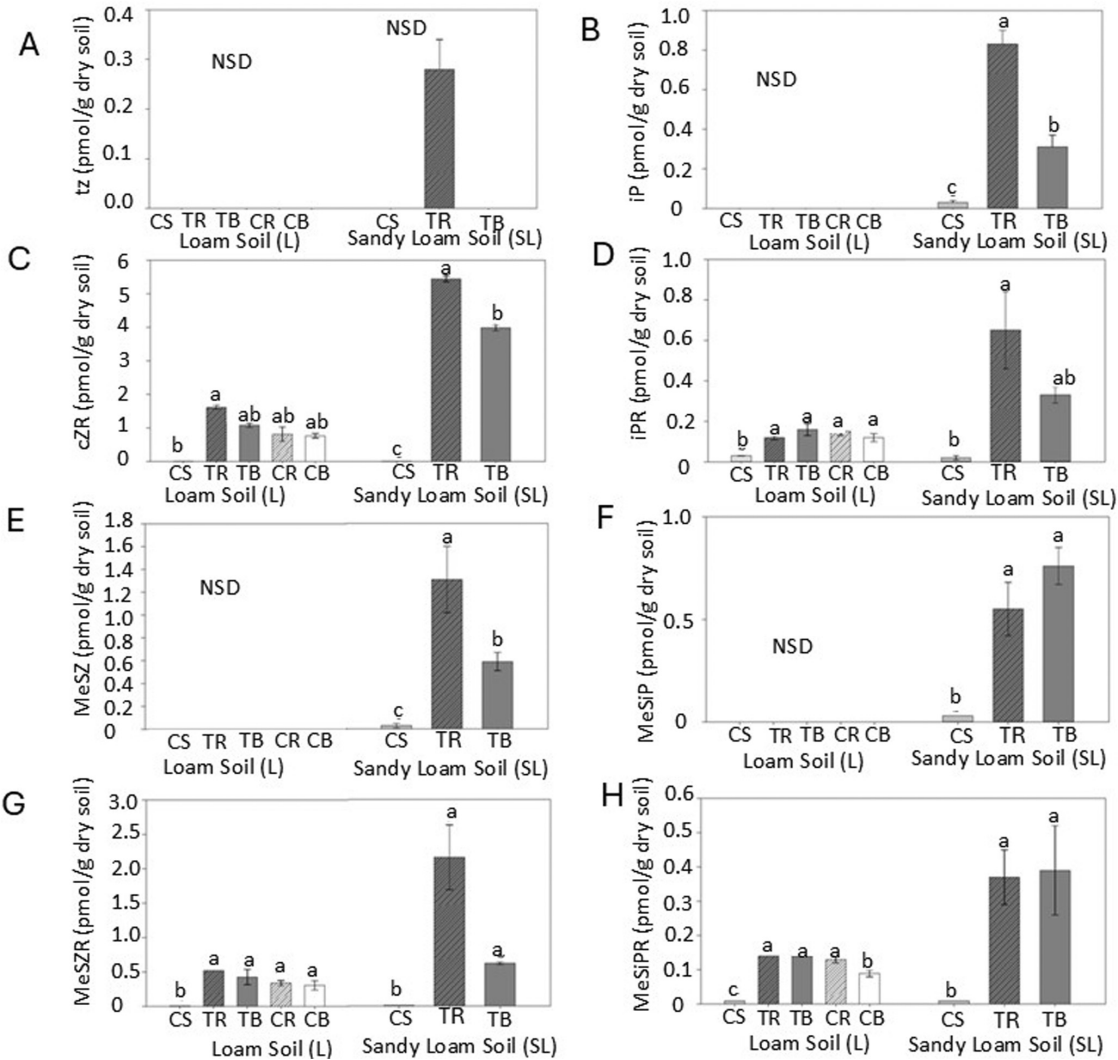
Effect of cover crops on CKs concentrations (pmol g^−1^ dry weight) **(A)** *trans*‐zeatin (tZ), **(B)** Isopentenyladenine (iP), **(C)** *cis*‐Zeatin riboside (cZR), **(D)** Isopentenyladenosine (iPR); **(E)** 2‐methylthio‐zeatin (2MeSZ), **(F)** 2‐Methylthio‐N6‐isopentenyladenine (2MeSiP), **(G)** 2‐Methylthio‐zeatin riboside (2MeSZR), **(H)** 2‐Methylthio‐N^6^‐isopentenyladenosine (2MeSiPR), compared to control, uncultivated soils. ANOVA was used to compare metrics between treatments separately in loam soil and sandy loam soil. Values are mean ± SE (*n* = 3), different letters above columns indicate significant difference among treatments (Fisher's least significant difference test, *P* ≤ 0.05). control soil (CS); tillage radish, rhizosphere soil (TR); tillage radish, bulk soil (TB); cereal rye, rhizosphere soil (CR); cereal rye, bulk soil (CB).

Among the 32 CK types scanned using HPLC‐HRMS/MS, four CK forms were detected in L soils while eight CK forms were detected in SL soils. Moreover, cover‐cropped SL soils had higher levels of the detected CKs, compared to cover‐cropped L soils (Fig. 6A–H). Free‐base tZ and iP CKs were detected only in SL soil samples. The most active CK form, tZ, was found only in SL‐TR, while no tZ was detected in SL‐TB or SL control soils (Fig. 6A). Furthermore, levels of iP were 10‐fold higher in SL‐TR soil compared to SL control soils (Fig. 6A,B). Riboside CKs (cZR and iPR) were present in both soil types (L and SL). Notably, cZR was the most abundant CK form detected in this study. Both cZR and iPR were significantly elevated in both zones of cover‐cropped soils compared to the respective control soils (Fig. 6C,D).

Four forms of 2MeS‐CKs (2MeSZ, 2MeSZR, 2MeSiP, and 2MeSiPR) were found in SL soils, and two forms (2MeSZR and 2MeSiPR) in L soils (Fig. 6E–H). In general, profiles of 2MeS‐CKs resembled those of their free base‐CK and RB‐CK conjugates, both in patterns and abundance. Namely, 2MeSZ and 2MeSiP was found only in SL soils, while 2MeSZR and 2MeSiPR was detected in both soil types. When detected, 2MeS‐CKs levels were significantly higher in both soil zones compared to the control soils. In tillage radish SL soils, 2MeSZ and 2MeSZR levels were significantly different between soil zones, where rhizosphere soils had higher 2MeSZ and 2MeSZR levels compared to their bulk soils (Fig. 6E, G). In cereal rye L soils, 2MeSZR and 2 MeSiP levels were not significantly different between soil zones. (Fig. 6F, H).

### Relationship between AC, SMC diversity, EEA, and total CKs in cover‐cropped soils

**Fig. 7:**
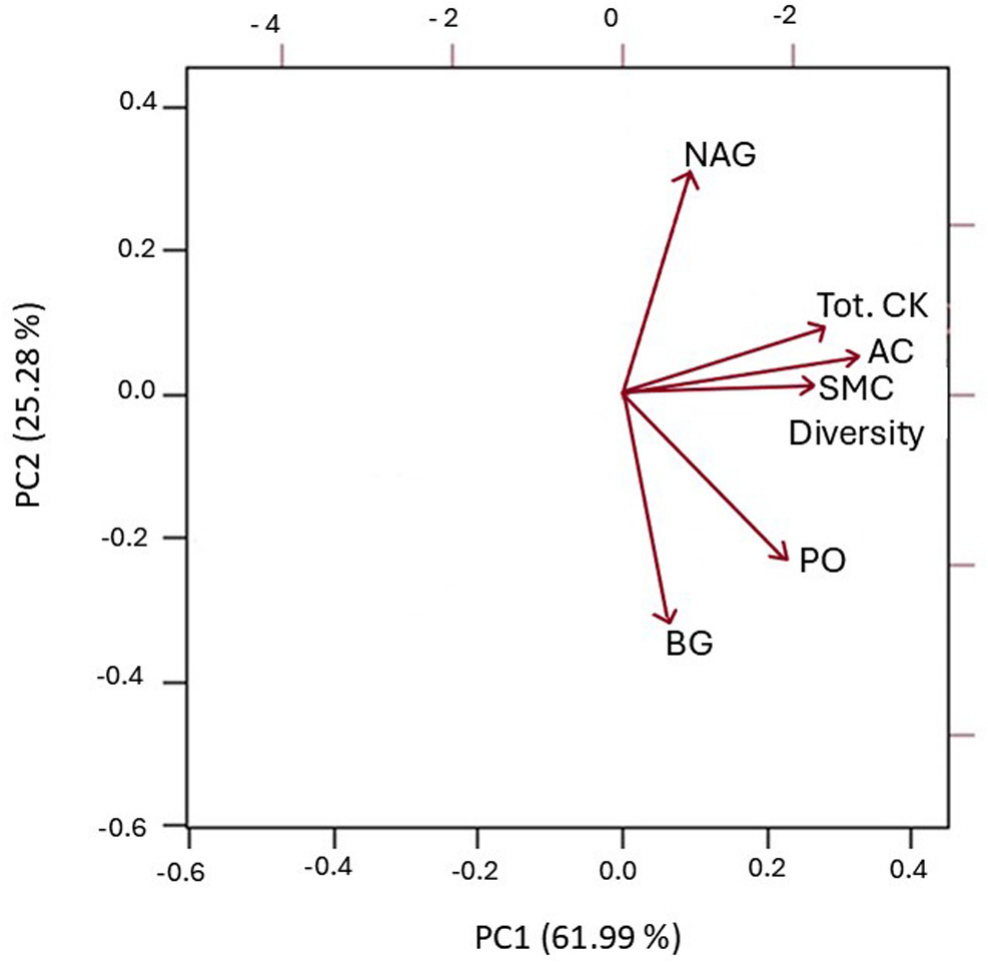
2D representation of PC1 and PC2 derived from the principal components analysis (PCA) of the dataset total cytokinins (Tot. CK), active carbon (AC), soil microbial community (SMC) diversity, and enzyme activity of ꞵ‐glucosidase (BG), phosphatase (PO), N‐acetylglucosaminidase (NAG) in loam rhizosphere and in bulk soil samples under the cover crops tillage radish and cereal rye.

An sPCA was conducted to visualize the multivariate relationships among soil health parameters (SMC diversity, AC content, EEA) and total CK levels in different soil types/zones/cover crop (Fig. 7). The first two principal components, PC1 and PC2, accounted for 87.27% cumulative variance. Active C content and SMC diversity loaded on PC1, showing a strong correlation with total CK abundance, while EEAs loaded on PC2.

## DISCUSSION

Cover cropping is a sustainable agricultural management strategy used to protect soil from erosion and improve soil health during the fallow period. Cover cropping is directly linked with an increase in SOM levels and can be used as a management strategy to restore SOC lost from agricultural lands (Lal 2004; Olson *et al*. 2010; Kaspar & Singer 2015). Management that increases SOM improves soil health via retention of water and nutrients, reduction of soil erosion, improvement of soil structure, facilitation of good drainage and aeration in soils, and subsequent improvement in crop yields (Lal 2006; Olson *et al*. 2010; Musinguzi *et al*. 2015; Oldfield *et al*. 2019). In our study, significantly higher SOM levels were observed in the CR loam (L) soil compared to the control (uncultivated) L soil (Fig. 2A). Moreover, we found significantly higher levels of AC in each type of cover‐cropped L soil, as well as in the TR samples collected from the sandy loam (SL) soil (Fig. 2B), revealing the positive impact of living roots on soil health. Furthermore, our results clearly indicate the positive influence of the proximity of living roots on AC levels, as we observed higher levels of AC in the rhizosphere compared to the bulk soils (Fig. 2B). This likely reflects increased C inputs from rhizodeposition and root exudation (Mutegi *et al*. 2011).

Active C, which is the labile fraction of SOC, represents substrate for SMCs, and is one of the major limiting factors for SMC growth (Hargreaves & Hofmockel 2014). Therefore, C added via living roots improves substrate availability for the growth of SMC (Hargreaves & Hofmockel 2014). The higher SMC diversity observed in the rhizosphere soils compared to the bulk soils is most likely related to large, diverse inputs of plant‐derived C into rhizosphere soil. The composition, quantity, and quality of C entering the soil via root exudates and plant detritus depend on the plant species, plant productivity, and fluctuations in abiotic factors; in turn, the quality and quantity of these inputs impact the diversity, composition, and abundance of the SMC (Hooper *et al*. 2000). Although previous studies reported changes in SMC diversity with plant species, no clear variation in SMC diversity was observed between the soils under the two cover crop species in our study (Fig. 3). Heterotrophic SMCs largely acquire the energy through the decomposition of organic material, which involves the conversion of complex organic matter into plant available nutrients via microbial EEAs, which play a major role in the cycling of C through the ecosystem (Six *et al*. 2006). Therefore, the utilization of C sources by SMCs represents a critical process in the cycling of C in the ecosystem (Lladó & Baldrian 2017). Community‐level physiological profiling reveals the functioning of SMC in the ecosystem based on utilization of substrate (C sources) (Lladó & Baldrian 2017). Our CLPP data showed a divergent trend in C source utilization by SMCs between soil zones, reflecting the impact of living roots and their associated root exudates on the metabolic functioning of SMCs in the rhizosphere (Fig. 4).

Microbial EEAs are important soil health parameters that reflect the functioning of the soil microbiome (Six *et al*. 2006). Microbial enzymes are responsible for the degradation of complex SOC to simple C compounds, which heterotrophic SMCs use to fulfil their energy needs. The type and the quantity of enzymes released by SMC depend primarily on the composition and abundance of substrates available in the environment (Six *et al*. 2006). In our study, the observed variation in NAG activity between cover crop types indicates that plant species may affect the composition and abundance of C sources available for microbial degradation.

The analysis of the traditional soil health parameters in our study provided further evidence of the important role of living roots in improving soil health. Plants can alter their environment via the secretion of root exudates (Huang *et al*. 2014). Root exudates are rich in C‐based organic compounds, including amino acids, organic acids, sugars, phenolics, proteins, and secondary metabolites, as well as inorganic ions, inorganic acids, water, and oxygen (Reviewed in Badri & Vivanco 2009). In addition to these compounds, a few early studies suggested the presence of CK phytohormones (free base‐CKs (iP, cZ, tZ), riboside‐CKs (cZR, tZR), and conjugated‐tZ (glucosides and nucleotides)) in rice root exudates (Murofushi *et al*. 1983; Soejima *et al*. 1992), which provided the compelling suggestion that plants can release endogenous CKs to the outside rhizosphere environment. Aligning with this claim, we detected significantly higher levels of total CKs in both zones of soils under both tillage radish and cereal rye compared to the control soils (Fig. 5). Furthermore, CK levels were higher in rhizosphere soils compared to bulk soils, emphasizing the fact that living roots, and perhaps associated microorganisms, are probable sources of CKs in soil.

Many previous studies reported the presence of free base CKs and riboside CKs in the roots of plants such as bean (*Phaseolus vulgaris*) (Allee and Republic, 1981), pea (*Pisum sativum* L.) (Short & Torrey 1972), maize (*Zea mays* L.) (Takei *et al*. 2001; Zalabák *et al*. 2014), wheat (*Triticum aestivum* L.) (Kudoyarova *et al*. 2014), soybean (*Glycine max*) (Prudent *et al*. 2016), ryegrass (*Lolium multifolorum*) (Wang *et al*. 2012; Guo *et al*. 2020), tomato (*Solanum lycopersicum*) (Glanz‐Idan *et al*. 2020), potato (*Solanum tuberosum* L.) (Raspor *et al*. 2020), and in other plant parts, such as seeds of soybean (Kambhampati *et al*. 2017), annual ryegrass (*Lolium rigidum*) (Goggin *et al*. 2015) or barley (*Hordeum vulgare* L.) (Powell *et al*. 2013). Hewett & Wareing (1973) showed the presence of high CK levels in senescing leaves, emphasizing the role of degrading plant matter in secreting CKs into the soil matrix. Although previous studies have detected only free base CKs (iP, tZ, cZ), RB‐CKs (cZR, tZR, iPR) and conjugated tZ in root exudates (Murofushi *et al*. 1983; Soejima *et al*. 1992), the majority of studies that found CKs in roots suggested that roots secrete CKs accumulated and synthesized inside the roots into the soil environment via root exudation (Arthur *et al*. 2001). The evidence indicates that plants may significantly contribute to pools of CKs in soil. These findings also support the claim that at least some of the CKs detected in our study are likely of plant origin. In our study, 2MeS‐CKs were among the most dominant forms of CKs in the soil samples (Fig. 6E–H). It is possible that the 2MeS‐CKs detected in rhizosphere soils could be of microbial origin. The methylthiolated CKs are thought to be synthesized in microorganisms in higher abundance compared to other life forms (Reviewed in Gibb *et al*. 2020). The rhizosphere is rich in the abundance and diversity of SMCs (estimated at 10^11^ microbial cells g^−1^ plant roots) and the rhizosphere microbiome is often considered to represent the plant's “second” genome (Egamberdieva *et al*. 2008; Berendsen *et al*. 2012).

While the classical measures of soil health (SOM, AC, SMC diversity) responded to cover cropping in our trials as predicted, soil CK profiles showed more dynamic and indicative changes among all conditions and situations for which better soil health would be expected, e.g., soils with living roots. This may reflect the combined contributions of both plants and SMCs to overall soil health. For example, both zones of the cover‐cropped soils had considerably higher CK levels compared to control soils. This may reflect a useful “half‐life” of CK existence in soil, whereby they are produced by living organisms but rapidly disappear in soils without living roots as some remaining organisms utilize the remaining CKs, after CK production stops. The relatively short persistence of CKs in soil may be explained, at least partially, because of the energy and nutrient‐rich nature of CK molecules, which microbes could quickly take up and use directly, or use within the purine salvage pathway (Ashihar *et al*. 2018). As such, the expected duration of CK presence in soil is likely to be relatively short and may partially rely on the presence of additional organisms associated with plants that are capable of producing and releasing new CKs to the soil (e.g., bacteria, fungi, protists, nematodes, insects) (Kisiala *et al*. 2013; Aoki *et al*. 2019; Andreas *et al*. 2020).

Beyond the significant differences in CK concentrations observed between cover crop soils and control soils, even more subtle distinctions were observed among cover crop treatments. For example, rhizosphere zones generally had higher CK levels than bulk soil zones for both tillage radish and cereal rye. Known as the most active CK, tZ was only detected in TR, while another active free base‐CK form, iP, was more widely detected, and the concentration increased in the order of TR > TB > control soil. Riboside‐CK profiles can be used as a proxy for what is happening with more active free base CKs, since they are often detected in higher abundances – although their activity level is still a matter of debate (Nguyen *et al*. 2021; Romanov & Schmülling 2021). According to the profiles of cZR and iPR detected in our work, these CKs were the most common forms found in the analysed samples and were highly variable among cover crops and soil zones (Fig. 6C, D). As such, they are potentially a strong indicator of short‐term changes in soil health. Similarly, 2MeS‐CKs were consistently detected at relatively high levels in soils (Fig. 6E–H). The challenge with 2MeS‐CKs is that their biological functions in plants or inn other organisms are still largely unknown (Gibb *et al*. 2020). However, to a large extent, 2MeS‐CK profiles seemed to mirror free base CKs and riboside CKs profiles and fluctuate in correspondence with soil health indicators.

As CKs are one of the critical growth‐promoting and yield‐enhancing agents, our CK results create a firm starting point for the potential use of soil CK profiles as new, sensitive parameters to assess the impact of agricultural management strategies on soil health in the short term. The positive associations observed in the conducted PCA analysis indicate strong connections between soil total CK levels and AC and SMC diversity (Fig. 7), which further emphasizes the potential.

Given the promise shown by our greenhouse trials, performed in pots with field‐collected soils, there is impetus to take new experiments into field plots. This will advance studies towards assessing the feasibility of using CKs as a soil health tool under practical conditions for growers. Comprehensive measures of CKs should be further explored to develop CKs as a novel soil health indicator to enable accurate assessments of the impact of agricultural management strategies on soil health in the short term, e.g., over a growing season. One such powerful new tool could be electrochemical sensors for CK detection, which could be adapted from the currently available approaches (Kim & Lee 2022) to measurements of liquid soil suspensions. This would allow real‐time, non‐destructive CK measurements for sensitive and rapid characterization of soil health.

In conclusion, the use of tillage radish and cereal rye cover crops resulted in a significant increase in soil health parameters, including SOM, AC, and SMC diversity, compared to soils without cover crops. In addition to evaluating these parameters, we purified CKs from the complex soil matrix using solid phase extraction and quantified them using HPLC‐HRMS/MS, which allowed identification and quantification with high specificity and sensitivity of eight CK forms. We observed strong associations between soil health indicators and total CKs (tZ, iP, cZR, iPR, 2MeSZ, 2MeSZR, 2MeSiP, 2MeSiPR). The assessed soil CK concentrations increased significantly in the cover‐cropped soils, suggesting the potential application of CK analysis as a tool to evaluate soil health as influenced by agricultural management strategies. Interestingly, total soil CK profiles showed a positive correlation with AC and SMC diversity, further supporting our suggestion that CK can be used as a soil health parameter. This is the first study reporting comprehensive soil CK analysis and the potential role of soil CK profiles as a novel, reliable soil health parameter.

## Author contributions

Conceptualization: IP, KAT, RJNE; Methodology: AK, IP, KAT, RJNE; Investigation: IP, KAT, RJNE; Data curation: IP, KAT, AK; Writing—original draft preparation: IP; Revisions: IP, KAT, RJNE, AK.

## Supporting information


**Table S1.** The guilds and names of 31 different carbon sources inside the wells of Biolog®EcoPlate™ (Fra̧c et al., 2012).
