# Supplementary material for: Soil health improvements under cover crops are associated with enhanced soil content of cytokinins
Source: Plant Biol (Stuttg). 2024 Dec 6;27(2):265–78. doi: 10.1111/plb.13743 (PMC11846634; doi:10.1111/plb.13743)
Supplement: Supplementary file 1 — Table S1. The guilds and names of 31 different carbon sources inside the wells of Biolog®EcoPlate™ (Fra̧c et al., 2012). [file PLB-27-265-s001.docx]

**Supplementary Table 1**

The guilds and names of 31 different carbon sources inside the wells of Biolog®EcoPlate™ (Fra̧c et al., 2012)

| **Guilds** | **Abbreviations** | **Name of the C-source** |
| --- | --- | --- |
| Amines & Amides | A1 | Phenylethylamine |
|  | A2 | Putrescine |
| Amino Acids | AA1 | L-Arginine |
|  | AA2 | L-Asparagine |
|  | AA3 | L-Phenylalanine |
|  | AA4 | L-Serine |
|  | AA5 | L-Threonine |
|  | AA6 | Glycyl-L-Glutamic Acid |
| Carbohydrates | C1 | Pyruvic Acid Methy Ester |
|  | C2 | D-Cellobiose |
|  | C3 | Alpha-D-Lactose |
|  | C4 | Beta-Methyl-D-Glucoside |
|  | C5 | D-Xylose |
|  | C6 | i-Erythritol |
|  | C7 | D-Mannitol |
|  | C8 | N-Acetyl-D-Glucosamine |
|  | C9 | Glusose-1-Phosphate |
|  | C10 | D,L-a-Glycerol Phosphate |
| Carboxylic & Acetic Acids | CA1 | D-Glucosaminic Acid |
|  | CA2 | D-Galactonic Acid y-Lactone |
|  | CA3 | D-Galacturonic Acid |
|  | CA4 | 2-Hydroxy Benzoic Acid |
|  | CA5 | 4-Hydroxy Benzoic Acid |
|  | CA6 | Gamma-Amino Butyric Acid |
|  | CA7 | Itaconic Acid |
|  | CA8 | alpha-Keto Butyric Acid |
|  | CA9 | D-Malic Acid |
| Polymers | P1 | Tween 40 |
|  | P2 | Tween 80 |
|  | P3 | Alpha-Cyclodextrin |
